# [Corrigendum] Hispolon inhibits breast cancer cell migration by reversal of epithelial‑to‑mesenchymal transition via suppressing the ROS/ERK/Slug/E‑cadherin pathway

**DOI:** 10.3892/or.2024.8793

**Published:** 2024-08-08

**Authors:** Zhao Zhao, Yi-Sheng Sun, Wei Chen, Long-Xian Lv, Yong-Quan Li

Oncol Rep 35: 896–904, 2016; DOI: 10.3892/or.2015.4445

Subsequently to the publication of the above paper, an interested reader drew to the authors' attention that, with the cell migration assay data shown in Fig. 7 on p. 901, the “TPA” and “TPA + U0126” panels were strikingly similar, such that data which were intended to show the results from differently performed experiments had apparently been derived from the same original source. In addition, it was noted that the “TPA + hispolon” and “TPA + NAC” data panels in Fig. 4B on p. 899 contained overlapping sections. Thirdly, a data panel was shared between Figs. 1 and 4, although this was intentional on the part of the authors as the same experiment was being portrayed in these figures.

The authors were able to re-examine their original data files, and realized that errors were made in asssembling Figs. 4B and 7. The revised versions of Figs. 4 and 7, now containing the correct data for the “TPA + NAC” experiment in Fig. 4B and the Control (“Ctrl”) experiment in Fig. 7, are shown on the next two pages. The authors wish to emphasize that the corrections made to these figures do not affect the overall conclusions reported in the paper, and they are grateful to the Editor of *Oncology Reports* for allowing them the opportunity to publish this corrigendum. All the authors agree to the publication of this corrigendum, and also apologize to the readership for any inconvenience caused.

## Figures and Tables

**Figure 4.**
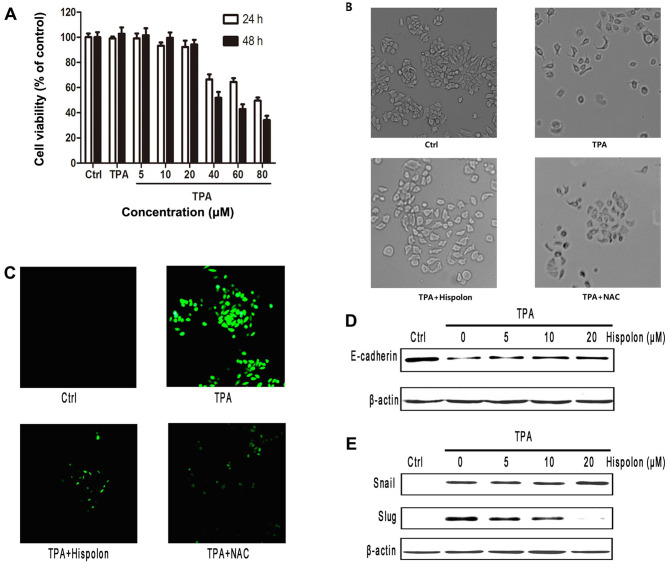
Hispolon inhibits TPA-induced EMT by reducing cellular ROS production at sublethal doses. (A) Effect of hispolon on the viability of MCF-7 cells. Following 24 h treatment with different doses of hispolon in the presence or absence of 100 ng/ml TPA, viability of MCF-7 cells was determined using MTT. Data represent mean ± SD of three independent experiments. (B) TPA-mediated morphological changes were abolished by hispolon. Cells were pretreated with hispolon (20 µM) or NAC (40 mM) for 3 h, followed by treatment with 100 ng/ml TPA for 24 h, and the resultant morphology was examined microscopically. (C) TPA-induced intracellular ROS production was inhibited by hispolon. MCF-7 cells were serum starved for 24 h and were pretreated with hispolon or NAC for 3 h, followed by treatment with 100 ng/ml TPA for 5 min. Cells were washed twice with PBS and fixed with 10% formaldehyde. Intracellular ROS levels were measured by staining with DCFH-DA (10 µM), and images were captured using a fluorescence microscope. (D) TPA-mediated downregulation of E-cadherin was abolished by hispolon. Cells were exposed to different doses of hispolon for 3 h and then were stimulated with 100 ng/ml of TPA for 24 h. E-cadherin protein levels were examined by western blotting. (E) Cells were pretreated for 3 h with hispolon, followed by treatment for 3 h (Snail) or 12 h (Slug) with 100 ng/ml TPA, and protein levels were analyzed by western blotting.

**Figure 7.**
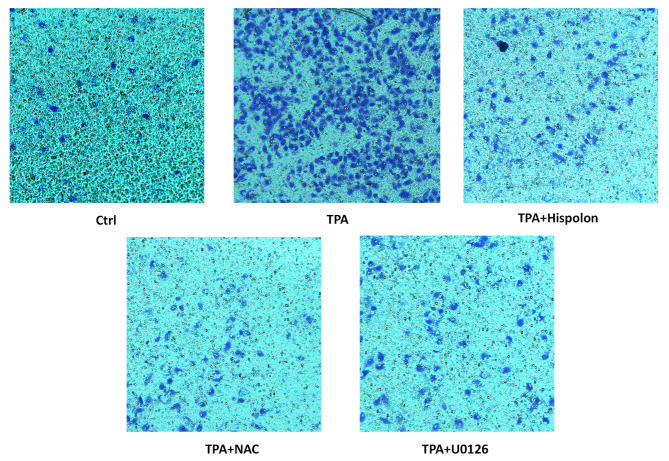
Hispolon inhibits TPA-induced cell migration. Cells were pretreated for 3 h with hispolon, NAC, U0126 and seeded into Transwell inserts and cultured by treatment with 100 ng/ml TPA for 24 h. The migrating cells were stained with crystal violet, and the resultant morphology was examined microscopically. All the data presented similar results from three independent experiments..

